# A Multi-Attribute Decision Method under Uncertainty Environment Conditions—The Green Supplier Evaluation Perspective

**DOI:** 10.3390/ijerph18010344

**Published:** 2021-01-05

**Authors:** Hao Xu, Liuxin Chen, Qiongfang Li, Jianchao Yang

**Affiliations:** 1 Business School, Hohai University, Nanjing 211100, China; lindawuge@hotmail.com (L.C.); scuyangjc@163.com (J.Y.); 2 College of Hydrology and Water Resources, Hohai University, Nanjing 210098, China; qfli@hhu.edu.cn

**Keywords:** hesitantly fuzzy TOPSIS, variance fluctuation, uncertainty, green suppliers

## Abstract

Due to the continuous changes of political environment, consumption habits, technological progress and other factors, the external environment of enterprises is full of uncertainty. The turbulence of external environment is not conducive to the long-term operation and development of enterprises, but also brings great challenges to the selection of suppliers. This makes the competition of enterprises focus on how to choose long-term cooperation suppliers in the uncertain external environment. In addition, due to the deterioration of the global environment, governments pay more and more attention to environmental pollution, and consumers are more and more inclined to green consumption, which makes many companies pay more and more attention to environmental indicators when selecting suppliers. In the case of external environment turbulence and serious environmental pollution, the evaluation and selection of green suppliers in uncertain environment is particularly important for the long-term development of enterprises. What’s more, when the supplier’s capability gap is small, the decision-maker often hesitates among several suppliers. In this paper, the hesitant fuzzy is used to describe the hesitant psychology of decision-makers in selecting suppliers, the variance fluctuation is used to describe the characteristics of hesitant fuzzy numbers, and the probability is used to measure the uncertainty of the environment. A green supplier evaluation model under the uncertainty environment is proposed, which comprehensively evaluates the green suppliers under the uncertain environment. Furthermore, it is compared with other methods that do not consider the uncertainty and the adaptability of evaluation method and right confirmation method, so as to reflect the influence of uncertainty to green supplier evaluation and the importance of adaptability of evaluation method and right confirmation method.

## 1. Introduction

With the acceleration of the economic globalization process, the external environment has an increasing impact on enterprises. In the context of economic globalization, companies must have the ability to respond to the external environment changes in order to develop steadily and be able to make judgments and respond to the environment in a timely manner. Therefore, environmental uncertainty plays a vital role in supplier evaluation. In order to further improve the accuracy of supplier evaluation, it is of great practical significance to incorporate environmental uncertainty into the supplier evaluation process.

Supplier selection has always been the focus of academic attention. At present, much research has been done on supplier evaluation, including innovation of evaluation methods, construction of evaluation factor system, etc. A lot of research work has been done on the supplier evaluation index system [1,2,3], and with the continuous development of economy and society, the supplier evaluation index is also changing. Dicson et al. obtained 23 supplier evaluation indicators through the survey of professionals in the procurement industry, and pointed out the eight most important indicators, which laid the foundation for the study of supplier evaluation indicators [4]. In the 21st century, people think that quality is an important index of supplier evaluation, while the importance of price, delivery ability and service is relatively weakened [5]. In recent years, as people’s awareness of environmental protection has increased, people have begun to pay attention to the importance of environmental indicators in supply chain evaluation and have come to believe that environmental indicators should also be integrated into supply chain management, which is conducive to the long-term stable development of enterprises [6,7]. In order to select better suppliers many evaluation methods are used. Some studies use the linear programming method to evaluate suppliers from the perspective of supplier performance [8,9]. Some scholars think that the pairwise comparison method can evaluate suppliers comprehensively and select the most suitable suppliers [10,11,12]. There are also some studies to solve the problem of supplier evaluation and selection from the perspective of value or utility [13,14,15].

Green supply chains can avoid the waste of resources, enhance the sense of social responsibility of enterprises [16,17], bring good reputation and brand image of green products to enterprises, and expand the product market [18]. As an important part of the supply chain, the evaluation of green suppliers is being paid more and more attention by enterprises. In recent years, due to the deterioration of the environment, the evaluation and selection of green suppliers have attracted more and more attention. Quan et al. used the weighted grey decision model to select the green suppliers of processing and manufacturing enterprises [19]; Liu et al. proposed a two-stage comprehensive evaluation method of green suppliers for green fresh products [20]; Liou et al. proposed a data-driven green supplier evaluation model on the basis of random forest algorithm [21]; Dobos et al. used data envelope analysis to evaluate green suppliers in the case of incomplete data [22]. Fallahbour et al. evaluated green suppliers based on fuzzy theory and carbon management standards [23]; Qu et al. proposed a random dual fuzzy language to rank green suppliers based on group satisfaction and regret theory [24]; Gao et al. put forward a group formula decision model when selecting green suppliers for the electronics manufacturing industry [25]. The evaluation of green suppliers is a strategic decision-making problem for the company, whose purpose is to ensure the green and efficient supply chain of enterprises. The purpose of the evaluation of green suppliers is to make the best choice while ensuring environmental protection to obtain the greatest benefits.

With the advancement of global economic integration, the business environment of enterprises is facing greater uncertainty [26] and environmental uncertainty will lead to business risks, performance fluctuations and information asymmetry, which will directly affect the financing cost and financing difficulty of listed companies in the external capital market [27]; Wang et al. found that environmental uncertainty directly affects marketing knowledge process management capabilities through investigation and research [28]; Chen et al. stated that the dynamics and complexity of the environment can strengthen the positive relationship between strategic change and performance, and alleviate the negative relationship between them [29]. Sun independently calculated the uncertainty of the environment in different scenarios, so as to evaluate the energy-saving projects of machinery manufacturing enterprises in each scenario [30].

However, most of the above studies on supplier evaluation focus on the selection of evaluation methods and the establishment of evaluation indicators, ignoring the matching between the methods and the actual situation, and ignoring the effect of the uncertainty of the external environment of suppliers. The uncertainty of the environment and the adaptability between methods and method of right confirmation are very important for supplier evaluation and must be considered in the supplier evaluation process. Therefore, previous studies may not fully accurately reflect the evaluation results. In view of this, according to the psychological characteristics of decision-makers’ indecision in the selection process, this paper uses hesitant fuzzy theory to describe the issue, uses variance fluctuation to confirm weights according to the fluctuation characteristics of hesitant fuzzy numbers, and uses the relevant knowledge of probability theory to measure the uncertainty of the external environment of the enterprise, and finally combines the three factors to put forward a green supplier evaluation under uncertain environment conditions, to conduct a comprehensive and effective evaluation of suppliers. What’s more, the effectiveness of the model is proved by comparing the results with previous research.

## 2. Materials and Methods

Today’s world economic environment is full of uncertainty, so it is difficult to accurately evaluate green suppliers. For example: the turbulence of political environment, the change of consumption habits, the progress of science and technology and other uncertain factors will affect the evaluation of green suppliers. Therefore, the selection of evaluation methods should adapt to the uncertainty of the external environment. Only when the evaluation methods adapt to the uncertainty of the external environment can we make a comprehensive and effective evaluation of green suppliers. In the selection process of green suppliers, people will be hesitant in deciding between several or even more suppliers, especially when the strengths of several green suppliers are not far from each other, it is very difficult for decision makers to make a decision. In the process of supplier evaluation, the determination of attribute weight is only an important part of green supplier evaluation. The determination of attribute weight should adapt to the evaluation and selection method of green suppliers, whereby the adaptation of confirmation methods and evaluation methods will produce better evaluation results. In view of the problems existing in the process of supplier evaluation, this paper selects hesitant fuzzy theory to describe the phenomenon of decision-makers’ hesitation among several choices when making decisions, and describes the fluctuation of hesitant fuzzy numbers with the method of confirming weights based on variance fluctuation, so as to improve the adaptability of weight determination method and evaluation method and describe the change of enterprise external environment with uncertainty analysis so as to evaluate green suppliers comprehensively and effectively.

### 2.1. Hesitation Fuzzy

In 1965, Zadeh first proposed the concept of fuzzy sets, which is used to express uncertain and fuzzy information [31]. Torra proposed using hesitant fuzzy sets in order to solve the problem of people’s indecision when making choices [32]. On the basis of hesitant fuzzy sets, Zhu et al. proposed dual hesitant fuzzy sets considering non-membership degree [33]. As an important tool in the field of multi-attribute decision-making, hesitant fuzzy theory has been further studied by many experts and scholars [34]. It is used in combination with many other related theories, such as TOPSIS and Choquet integrals [35]. Hesitant fuzzy theory is also widely used in many fields, such as risk factor analysis of emergencies [36]. In recent years, hesitation fuzzy theory has made great progress as a new description tool for uncertain decision information. Luo et al. took the risk preference of decision-makers into account and proposed a decision-making method for emergencies in the presence of risk factors [37]. Torra proposed the use of hesitating fuzzy sets for multi-attribute decision-making problems [32]; Bedregal et al. studied various integrated functions of hesitating fuzzy numbers from a mathematical perspective [38]; Farhadinia proposed the score function of hesitating fuzzy numbers and applied it to the ranking of hesitant fuzzy numbers [39]; Zhang et al. proposed a hesitant fuzzy QUALIFLEX decision-making method based on marked distance, which is used to solve multi-attribute decision-making problems in which the attribute value and attribute weight value are both hesitating fuzzy numbers [40]. Zhao et al. combined the definitions of hesitant fuzzy sets and triangular fuzzy numbers and proposed the concept of hesitant triangular fuzzy sets [41].

**Definition** **1.** *Let*X*be a non-empty set [32,42].*$H=\left\{\langle x,h_{H}\left(x\right)\rangle x\in X\right\}$*is the hesitation fuzzy set. Among them,*$h_{H}\left(t\right)$*is a set composed of different numbers on the interval [0,1], representing several possible membership degrees of elements*x∈H*in the set*X. $h_{H}\left(x\right)$*is the hesitation fuzzy number, expressed in detail as*$h_{H}\left(x\right)=H\left\{\gamma^{1},\;\gamma^{2},\;\gamma^{3}\cdots \gamma^{\#h}\right\}\left(\gamma^{\theta }\in \left[0,1\right],\theta =1,2,3,\cdots ,\#h\right)$*, where*#h*represents the number of elements in the hesitation fuzzy number*$h_{H}\left(x\right)$*, that is the maximum value of*θ*. If*#h=1*, then the hesitation fuzzy set*H*will degenerate into a traditional fuzzy set.*

**Definition** **2.** *For any three hesitant fuzzy numbers* $h_{H}{\left(x\right)}_{1}$ *,* $h_{H}{\left(x\right)}_{2}$ *and* $h_{H}{\left(x\right)}_{3}$ *, the basic algorithm are as follows [32,42]:*

(1) $h_{H}{\left(x\right)}_{1}\cup h_{H}{\left(x\right)}_{2}=H\left\{\max\left(\gamma_{1},\gamma_{2}\right)|\gamma_{1}\in h_{H}{\left(x\right)}_{1},\gamma_{2}\in h_{H}{\left(x\right)}_{2}\right\}$;
(2) $h_{H}{\left(x\right)}_{1}\cup h_{H}{\left(x\right)}_{2}=H\left\{\min\left(\gamma_{1},\gamma_{2}\right)|\gamma_{1}\in h_{H}{\left(x\right)}_{1},\gamma_{2}\in h_{H}{\left(x\right)}_{2}\right\}$;
(3) $\theta h=H\left\{\left(1-{\left(1-\gamma \right)}^{\theta }\right)|\gamma \in h_{H}\left(x\right)\right\}\left(\theta >0\right)$;
(4) $h^{c}=H\left\{\left(1-\gamma \right)|\gamma \in h_{H}\left(x\right)\right\}$;
(5) $h^{\theta }=H\left\{\gamma^{\theta }|\gamma \in h_{H}\left(x\right)\right\}\left(\theta >0\right)$;
(6) $h_{H}{\left(x\right)}_{1}⊕h_{H}{\left(x\right)}_{2}=H\left\{\left(\gamma_{1}+\gamma_{2}-\gamma_{1}\gamma_{2}\right)|\gamma_{1}\in h_{H}{\left(x\right)}_{1},\gamma_{2}\in h_{H}{\left(x\right)}_{2}\right\}$;
(7) $h_{H}{\left(x\right)}_{1}⊗h_{H}{\left(x\right)}_{2}=H\left\{\gamma_{1}\gamma_{2}|\gamma_{1}\in h_{H}{\left(x\right)}_{1},\gamma_{2}\in h_{H}{\left(x\right)}_{2}\right\}$;

**Definition** **3.** *In the actual decision-making process, the number of elements in different hesitant fuzzy numbers may be different and disordered, which will make it very difficult to deal with two fuzzy numbers. For the convenience of calculation and without loss of generality, Xu and Xia suggested that the fuzzy number should be standardized before calculation [43].*

First, we arrange all elements in the hesitation fuzzy number in increasing order, for example: turn $h_{H}\left(x\right)=H\left\{0.9,0.1,0.5,0.2\right\}$ into $h_{H}\left(x\right)=\left\{0.1,0.2,0.5,0.9\right\}$. Secondly, for any two hesitant fuzzy numbers $h_{H}{\left(x\right)}_{1}$ and $h_{H}{\left(x\right)}_{2}$, when the number of their elements is not the same, that is $\#h_{1}\neq \#h_{2}$, Xu and Xia give two extension rules.

First, if the decision maker is risk-averse, we increase the number of smallest elements in fuzzy numbers until the number of elements in all hesitation fuzzy numbers is the same. Second, if the decision maker is risk preferent, we increase the number of largest elements in fuzzy numbers until the number of elements in all hesitation fuzzy numbers is the same.

Based on the two extension rules proposed by Xu and Xia, Zhang proposed an extension method with parameters [44], that is for hesitant fuzzy numbers $h_{H}\left(x\right)=H\left\{\gamma^{\lambda }|\lambda =1,2,3,\cdots ,\#h\right\}$, let $\gamma^{+}$ and $\gamma^{-}$ be the largest and smallest elements in the hesitation fuzzy number $h_{H}\left(x\right)$ respectively, we call $\bar{\gamma }=\alpha \gamma^{+}+\left(1-\alpha \right)\gamma^{-}$ as an extended value with parameters, where the parameter α(0≤α≤1) is given by the decision maker according to his own risk preference. This method considers all the risk preferences of decision makers, such as risk preference, risk neutrality and risk aversion. When α=1 or α=0, they represent two special forms of risk preference and risk aversion respectively.

**Definition** **4.** *Xu and Xia proposed the measure distance between two hesitant fuzzy numbers* $h_{H}{\left(x\right)}_{1}$ *and* $h_{H}{\left(x\right)}_{2}$ *, it is represented by hesitant fuzzy Hamming distance measure and hesitant fuzzy Euclidean distance measure [45].*

*Hesitant fuzzy Hamming distance measure:* $d_{H}\left(h_{H}{\left(x\right)}_{1},h_{H}{\left(x\right)}_{2}\right)=\frac{1}{\#h}\sum_{\lambda 1}^{\#h}\left|\gamma_{1}^{\lambda }-\gamma_{2}^{\lambda }\right|$

*Hesitant fuzzy Euclidean distance measure:* $d_{E}\left(h_{1},h_{2}\right)=\sqrt{\frac{1}{\#h}\sum_{\lambda =1}^{\#h}{\left(\gamma_{1}^{\lambda }-\gamma_{1}^{\lambda }\right)}^{2}}$

### 2.2. Hesitant Fuzzy TOPSIS

The technology for order preference by similarity to ideal solution (TOPSIS) model, proposed by Hwang and Yoon in 1981, makes decisions according to the proximity of evaluation objects to rational solutions [46]. Xu et al. proposed a hesitant fuzzy multi-attribute decision-making method based on TOPSIS to solve decision-making problems with incomplete weight information [47]. The hesitant fuzzy decision-making method has been widely used. For example, Zhang et al. combined hesitant fuzzy with a generalized Choquet method to evaluate suppliers with risks [48]. Tan et al. defined the Hamming distance and Euclidean distance of intuitionistic hesitant fuzzy sets and proposed a TOPSIS decision-making method for intuitionistic hesitant fuzzy sets [49].

In the process of hesitant fuzzy multi-attribute decision-making, in many cases, it is necessary to resort to the ordering of hesitant fuzzy numbers. For multi-attribute decision-making problems where the attribute value is a hesitating fuzzy number, the element in the hesitation fuzzy number mean that the decision maker is hesitant in several evaluation values. The traditional method of determining weights cannot fully reflect the characteristics of the fuzzy number. Therefore, this paper introduces the weight determination method based on variance fluctuation to solve the particularity of hesitant fuzzy number. The process of using hesitating fuzzy TOPSIS to solve multi-attribute decision problems is as follows.

#### 2.2.1. Co-Trend Hesitation Fuzzy Matrix

Firstly, the standardized hesitation fuzzy decision matrix A of the sample data is established according to the decision information, and then the hesitation fuzzy matrix A is standardized according to the Definition 3 to obtain the standardized hesitation fuzzy matrix $\bar{A}$. Evaluation attributes can usually be divided into benefit type and cost type. In order to eliminate the influence of different index dimensions, ensure the compatibility between all attributes, and make full use of the information of the original data, the TOPSIS method requires all factors to change in the same direction when evaluating. We usually handle it like this: transform high-quality factors into low-quality factors, or convert low-quality factors into high-quality factors, that is co-trend. The standardized hesitation fuzzy matrix $\bar{A}$ is treated with the same trend to obtain the co-trend hesitation fuzzy matrix $\tilde{A}$. Using the method proposed by Zhu and Xu to transform cost-type attributes into benefit-type attributes [50], for benefit-type attributes ${\tilde{h}}_{ij}={\bar{h}}_{ij}$, for cost-type attributes ${\tilde{h}}_{ij}={\left({\bar{h}}_{ij}\right)}^{c}$, where ${\left(h_{ij}\right)}^{c}=H\left(1-\gamma_{ij}^{1},1-\gamma_{ij}^{2},\cdots ,1-\gamma_{ij}^{\#h}\right)$:

$$\tilde{A}=\left[\begin{array}{cccc} {\tilde{h}}_{11} & {\tilde{h}}_{12} & \cdots & {\tilde{h}}_{1n}\\ {\tilde{h}}_{21} & {\tilde{h}}_{22} & \cdots & {\tilde{h}}_{2n}\\ \vdots & \vdots & \ddots & \vdots \\ {\tilde{h}}_{m1} & {\tilde{h}}_{m2} & \cdots & {\tilde{h}}_{mn} \end{array}\right]$$

#### 2.2.2. Weight Determination Method Based on Variance Fluctuation

There are many methods to determine the weight, mainly divided into two categories: subjective methods and objective methods. Subjective weighting methods such as the AHP method, the Delphi method, etc., are mainly obtained by experts’ subjective judgment based on experience. These methods have been studied earlier and are more mature, but their objectivity is poor. The original data of an objective weighting method such as the variation coefficient method, the entropy weight method, etc., is composed of the actual data of each factor in the evaluation, so it does not depend on the subjective judgment of human beings, and this kind of method has strong objectivity. In the past, most of the weight determination methods paid attention to the subjective and objective aspects and less attention was given to the adaptability of the confirmation method and the evaluation method, ignoring the relationship between the two. The weight determination method adapts to the evaluation method, which will make the evaluation results more in line with the actual situation. Therefore, according to the particularity of evaluation method, variance fluctuation is used to fully reflect the characteristics of fuzzy number. The element of hesitant fuzzy number mean that the decision-maker is indecisive among several evaluation values, which has certain particularity. Accordingly, variance is a tool that can be to measure the fluctuation degree of a group of data, which accords with the characteristics of fluctuation between elements in hesitant fuzzy numbers. Therefore, in order to solve the particularity of hesitation fuzzy and the objectivity of evaluation, we use the method based on “variance fluctuation” to determine the weight:

(1) $$S_{k}=\sqrt{\frac{1}{\sum_{i=1}^{n}\#h_{i}}\sum_{i=1}^{n}{\left(\gamma_{ki}^{j}-\bar{\gamma_{k}}\right)}^{2},j=1,2,3,\cdots ,\#h}$$

$$S_{k}=\sqrt{\frac{1}{n}\left[\begin{array}{c} {\left(\gamma_{k1}^{1}-\bar{\gamma_{k}}\right)}^{2}+{\left(\gamma_{k2}^{1}-\bar{\gamma_{k}}\right)}^{2}+\cdots +{\left(\gamma_{k\#h}^{1}-\bar{\gamma_{k}}\right)}^{2}+{\left(\gamma_{k1}^{2}-\bar{\gamma_{k}}\right)}^{2}+{\left(\gamma_{k2}^{2}-\bar{\gamma_{k}}\right)}^{2}\\ +\cdots +{\left(\gamma_{k\#h}^{2}-\bar{\gamma_{k}}\right)}^{2}+{\left(\gamma_{k1}^{n}-\bar{\gamma_{k}}\right)}^{2}+{\left(\gamma_{k2}^{n}-\bar{\gamma_{k}}\right)}^{2}+\cdots +{\left(\gamma_{k\#h}^{n}-\bar{\gamma_{k}}\right)}^{2} \end{array}\right]}$$

where $\gamma_{ki}^{j}$ represents the j-th element in the i-th fuzzy number under the K-th factor, and $\bar{\gamma_{k}}$ represents the average number of all elements in all the fuzzy numbers under the k-th factor:

(2) $$W_{k}=\frac{S_{k}}{S_{1}+S_{2}+\cdots +S_{r}}$$

where $W_{k}$ represents the weight of the K-th factor, $S_{k}$ is the fluctuation between elements in the hesitation fuzzy number under the K-th factor, and r is the number of factors.

Multiplying the weight calculated based on the variance fluctuation to the right of the co-trend hesitation fuzzy matrix we obtain the weighted co-trend hesitation fuzzy matrix $\hat{A}$:

$$\hat{A}=\tilde{A}\cdot W=\left[\begin{array}{cccc} {\tilde{h}}_{11} & {\tilde{h}}_{12} & \cdots & {\tilde{h}}_{1n}\\ {\tilde{h}}_{21} & {\tilde{h}}_{22} & \cdots & {\tilde{h}}_{2n}\\ \vdots & \vdots & \ddots & \vdots \\ {\tilde{h}}_{m1} & {\tilde{h}}_{m2} & \cdots & {\tilde{h}}_{mn} \end{array}\right]\cdot \left[\begin{array}{cccc} w_{1} & 0 & \cdots & 0\\ 0 & w_{2} & \cdots & 0\\ \vdots & \vdots & \ddots & \vdots \\ 0 & 0 & \cdots & w_{n} \end{array}\right]=\left[\begin{array}{cccc} {\hat{h}}_{11} & {\hat{h}}_{12} & \cdots & {\hat{h}}_{1n}\\ {\hat{h}}_{21} & {\hat{h}}_{22} & \cdots & {\hat{h}}_{2n}\\ \vdots & \vdots & \ddots & \vdots \\ {\hat{h}}_{m1} & {\hat{h}}_{m2} & \cdots & {\hat{h}}_{mn} \end{array}\right]$$

#### 2.2.3. Determine the Optimal Evaluation Result

In the actual hesitant fuzzy multi-attribute decision-making problem, there is no hesitation fuzzy positive ideal solution and hesitant fuzzy negative ideal solution. Otherwise, the optimal solution of the multi-attribute decision-making problem is the hesitation fuzzy positive ideal solution, and the worst solution of the multi-attribute decision-making problem is the hesitation fuzzy negative ideal solution, so we use the hesitant fuzzy Euclidean distance to calculate the distance from each alternative to the hesitation fuzzy positive ideal solution and the hesitation fuzzy negative ideal solution, and then determine the best alternative.

First, we determine the hesitation fuzzy positive ideal solution $x^{+}$ and the hesitation fuzzy negative ideal solution $x^{-}$:

(3) $$x^{+}=\left\{c_{j},\max_{i=1}^{m}\langle {\tilde{\gamma }}_{ij}^{\lambda }\rangle |j=1,2,\cdots ,n.\lambda =1,2,\cdots ,\#h\right\}\;=\left\{c_{j},\tilde{h}\left(max{\tilde{\gamma }}_{ij}^{1},max{\tilde{\gamma }}_{ij}^{2},\cdots ,max{\tilde{\gamma }}_{ij}^{\#h}\right)|i=1,2,\cdots ,m.j=1,2,\cdots n.\lambda =1,2,\cdots ,\#h\right\}$$

(4) $$x^{-}=\left\{c_{j},\min_{i=1}^{m}\langle {\tilde{\gamma }}_{ij}^{\lambda }\rangle |j=1,2,\cdots ,n.\lambda =1,2,\cdots ,\#h\right\}\;=\left\{c_{j},\tilde{h}\left(min{\tilde{\gamma }}_{ij}^{1},min{\tilde{\gamma }}_{ij}^{2},\cdots ,min{\tilde{\gamma }}_{ij}^{\#h}\right)|i=1,2,\cdots ,m.j=1,2,\cdots n.\lambda =1,2,\cdots ,\#h\right\}$$

Then, we calculate the Euclidean distance $D\left(x_{i},x^{+}\right)$ and $D\left(x_{i},x^{-}\right)$ between each option and the positive and negative ideal solutions:

(5) $$D\left(x_{i},x^{+}\right)=\overset{n}{\underset{j=1}{\sum }}d_{E}\left({\tilde{h}}_{ij},{\tilde{h}}_{j}^{+}\right)=d_{E}\left({\tilde{h}}_{i1},{\tilde{h}}_{1}^{+}\right)+d_{E}\left({\tilde{h}}_{i2},{\tilde{h}}_{2}^{+}\right)+\cdots +d_{E}\left({\tilde{h}}_{in},{\tilde{h}}_{1}^{+}\right)\;=\overset{n}{\underset{j=1}{\sum }}\sqrt{\frac{1}{\#h}\overset{\#h}{\underset{\lambda =1}{\sum }}\left({\left(\gamma_{ij}^{1}-\gamma_{j}^{1+}\right)}^{2}+{\left(\gamma_{ij}^{2}-\gamma_{j}^{2+}\right)}^{2}+\cdots +{\left(\gamma_{ij}^{\lambda }-\gamma_{j}^{\lambda +}\right)}^{2}\right)}$$

(6) $$D\left(x_{i},x^{-}\right)=\overset{n}{\underset{j=1}{\sum }}d_{E}\left({\tilde{h}}_{ij,}{\tilde{h}}_{j}^{-}\right)=d_{E}\left({\tilde{h}}_{i1},{\tilde{h}}_{1}^{-}\right)+d_{E}\left({\tilde{h}}_{i2},{\tilde{h}}_{2}^{-}\right)+\cdots +d_{E}\left({\tilde{h}}_{in},{\tilde{h}}_{1}^{-}\right)\;=\overset{n}{\underset{j=1}{\sum }}\sqrt{\frac{1}{\#h}\overset{\#h}{\underset{\lambda =1}{\sum }}\left({\left(\gamma_{ij}^{1}-\gamma_{j}^{1-}\right)}^{2}+{\left(\gamma_{ij}^{2}-\gamma_{j}^{2-}\right)}^{2}+\cdots +{\left(\gamma_{ij}^{\lambda }-\gamma_{j}^{\lambda -}\right)}^{2}\right)}$$

Finally, we calculate the relative closeness $C_{i}$ of each option to the hesitation fuzzy positive ideal solution, and sort the options according to the relative closeness:

(7) $$C_{i}=\frac{D\left(x_{i},x^{-}\right)}{D\left(x_{i},x^{+}\right)+D\left(x_{i},x^{-}\right)}$$

### 2.3. Uncertainty Analysis

Most of the research on green suppliers focuses on the optimization of evaluation methods and the establishment of evaluation index systems [51,52], while less consideration is given to the uncertainty of the external environment in which the enterprise is located. However, the uncertainty of the external environment is very important for the selection of suppliers. The uncertainty of the external environment will not only affect the decision-making of the enterprise, but also affect the revenue of the enterprise [53].

For enterprises, uncertainty can be divided into two aspects, one is the dynamic environment faced by enterprises, the other is the complexity of the environment faced by enterprises. In the aspect of environmental complexity, it means that there are many external factors affecting the enterprise, such as political environment, economic environment, social environment, technological environment, etc., which will affect the enterprise’s supply chain system. In the aspect of environmental dynamics, it mainly refers to the instability of various elements in the environment, such as the number of suppliers, the instability of international relations, the speed of scientific and technological update iteration, the probability of economic crisis outbreak, etc., which will also affect the supply chain of enterprises. Uncertainty means that decisions must be made without obtaining sufficient information about the external environment, and it is difficult for decision makers to estimate changes in the external environment. The external environment of a company is a dynamic environment that is constantly changing, such as the promulgation of new laws, changes in market structure, changes in production processes caused by new technologies, changes in consumer tastes, etc. Different companies face different environments and the magnitude of uncertainty also varies depending on the external environment [54]. When the uncertainty faced by the company changes, the company will carry out flexible design, improve the forecasting level, optimize the inventory structure, and choose the transfer strategy, etc. to deal with the uncertainty, the implementation of the company’s internal strategy will change the company’s ability to respond to change [55].

Although the uncertainty of the environment will have a certain impact on the company, only when the change of the environment exceeds the company’s resilience will it have a negative impact on the company. The higher the environmental uncertainty, the greater the pressure the enterprise feels, and the more dynamic capabilities it will improve to deal with environmental uncertainty [56]. Specifically, in a hyper-competitive environment with high turbulence and fierce competition, companies are facing cruel competition, and competitors’ strategies and actions are unpredictable. Previous experience and even the experience of other companies in the same industry cannot help companies solve current problems. Instead, they must seek and explore new solutions to improve their dynamic capabilities. From a theoretical point of view, the company’s ability to respond to the environment will change with changes in the environment, which is a dynamic relationship, but it has a maximum. Based on the characteristics of this problem, we use the probability theory to indirectly estimate the impact of environmental uncertainty on the enterprise.

Let the response ability of the enterprise be $Z_{x}$ (the response ability in a certain environmental state), and the load ability is $Z_{a}$ (maximum response ability in the environment), and the state of the environment is $H_{y}$ (enterprises have different response abilities in different environments; load ability is the maximum response ability and is a fixed value, which depends on the strength of the enterprise itself).

Then the probability joint density function $f\left(Z_{x},H_{y}\right)$ of the enterprise’s respond ability $Z_{x}$ and environmental state $H_{y}$ is calculated as follows:

(8) $$f\left(Z_{x},H_{y}\right)=f\left(\frac{Z_{x}}{H_{y}}\right)f_{0}\left(H_{y}\right)$$

where $f\left(\frac{Z_{x}}{H_{y}}\right)$ is the conditional probability density function of $\;Z_{x}$ given a certain environmental state $H_{y}$; $f_{0}\left(H_{y}\right)$ is the probability density function of the environmental state. Using the total probability formula, we can get:

(9) $$f\left(Z_{x}\right)=\int_{-\infty }^{+\infty }f\left(\frac{Z_{x}}{H_{y}}\right)f_{0}\left(H_{y}\right)dH_{y}$$

Then the probability of an enterprise affected by the environment can be approximately expressed as:

(10) $$p=p\left(Z_{x}>Z_{a}\right)=\int_{Z_{x}}^{+\infty }\left[\int_{-\infty }^{+\infty }f\left(\frac{Z_{x}}{H_{y}}\right)f_{0}\left(H_{y}\right)dH_{y}\right]dZ_{x}\;=\int_{0}^{\infty }\left[\int_{Z_{x}}^{\infty }f\left(\frac{Z_{x}}{H_{y}}\right)dZ_{x}\right]f_{0}\left(H_{y}\right)dH_{y}$$

Let $F_{s}\left(H_{y}\right)=\int_{Z_{x}}^{\infty }f\left(\frac{Z_{x}}{H_{y}}\right)dZ_{x}$, then:

(11) $$p=\int_{0}^{\infty }F_{s}\left(H_{y}\right)f_{0}\left(H_{y}\right)dH_{y}\approx \int_{H^{*}}^{H**}F_{s}\left(H_{y}\right)f_{0}\left(H_{y}\right)dH_{y}$$

In the formula, $H^{*}$ represents the environmental state where the environment will affect the enterprise, and $H^{**}$ represents the extreme state of the enterprise affected by the environment. After discretization, the following results can be obtained

(12) $$p=\overset{n}{\underset{i=1}{\sum }}F_{s}\left(\bar{H_{i}}\right)\Delta F_{0}\left(\bar{H_{i}}\right)$$

where $F_{s}\left(\bar{H_{i}}\right)$ is the probability that the response ability $D_{s}$ is greater than the load ability $D_{r}$ for a given $H_{i}$, $\Delta F_{0}\left(\bar{H_{i}}\right)$ is the interval probability of the i section of the environmental state frequency curve, and n is the number of calculation segments of the environmental state frequency curve.

## 3. Example Analysis and Results

In recent years, governments have been advocating green development and consumers are advocating green consumption. At the same time, green production is brought about. With the improvement of people’s environmental awareness, more and more people begin to attach more importance to environmental issues and green development. In addition, in the environment of economic globalization, enterprises are affected by many external environmental factors, such as politics, economy, society, culture, technology, etc., and these external environments are highly uncertain. At the same time, the contradiction between economic development and environmental protection is becoming increasingly fierce, which further increases the uncertainty of today’s external environment. Therefore, in today’s era of advocating environmental protection and full of challenges, the evaluation of green suppliers has important theoretical significance and practical value.

### 3.1. Establishment of the Evaluation Factor System

There are many studies on supplier evaluation factor system, which are mainly focused on traditional factors such as price, quality, and supply capacity, etc. With the development of the economy and society, people are now paying more and more attention to green and low-carbon development, green and low-carbon are getting more and more attention from the government. The green factor is added base on the traditional evaluation factor in this paper.

With a background, a company intends to choose the best supplier from the four suppliers of $\left(x_{1},x_{2},x_{3},x_{4}\right)$. There are five main evaluation factors: cost factor $C_{1}$, quality level $C_{2}$, innovation capability $C_{3}$, environmental factor $C_{4}$, compatibility $C_{5}$. Construct the evaluation matrix of each supplier based on five main evaluation factors, as shown in Table 1.

### 3.2. Co-Trend Hesitation Fuzzy Matrix of Sample Data

First, establish a standardized hesitating fuzzy decision matrix of sample data according to the decision information. Then, the hesitation fuzzy matrix is standardized according to Definition 3, and the standardized hesitation fuzzy matrix is obtained. Finally, the method proposed by Zhu and Xu [50] is used to transform the cost-type attributes into profit-type attributes to perform co-trend processing on the standardized hesitation fuzzy matrix. The specific process is as follows:

According to the decision information, the hesitation fuzzy decision matrix of the sample data is constructed, as shown in Table 2.

The standardized hesitant fuzzy decision matrix is obtained by standardizing the hesitant fuzzy decision matrix, as shown in Table 3.

The standardized hesitant fuzzy decision matrix is processed with the same trend, and the co-trend hesitant fuzzy decision matrix is obtained, as shown in Table 4.

### 3.3. Weight Determination Method Based on Variance Fluctuation

The original data of the objective weighting method is composed of the actual data of each factor in the evaluation, which does not depend on the subjective judgment of human beings and has strong objectivity, so it is widely used in multi-attribute decision-making problems. The main characteristic of hesitant fuzzy multi-attribute decision-making problem is the particularity of hesitant fuzzy numbers, and the particularity of the hesitant fuzzy numbers is that the decision maker is hesitant between several evaluation values, which reflects the psychological fluctuation of the decision maker. In order to solve this characteristic of hesitating fuzzy numbers, this paper proposes a method of determining weight based on "variance fluctuation". The specific process is as follows:

According to the original data information in Table 2, for the hesitant fuzzy multi-attribute decision-making problem that the attribute weight $w=\left(w_{1},w_{2},w_{3},w_{4},w_{5}\right)$ is completely unknown, the variance fluctuation of hesitant fuzzy number under each attribute is calculated according to the method of determining the weight of “variance fluctuation”, and then the weight of each factor $w_{i}$ is calculated:

$$S_{k}=\sqrt{\frac{1}{\sum_{i=1}^{n}\#h_{i}}\sum_{i=1}^{n}{\left(\gamma_{ki}^{j}-\bar{\gamma_{k}}\right)}^{2}},j=1,2,3,\cdots ,\#h$$

so: $s_{1}=0.246$, $s_{2}=0.280$, $s_{3}=0.236$, $s_{4}=0.802$, $s_{5}=0.302$

$$W_{k}=\frac{S_{k}}{S_{1}+S_{2}+\cdots +S_{r}}$$

so: w=(0.132, 0.150, 0.126, 0.430, 0.162).

The attribute weight calculated based on the variance fluctuation method is multiplied by the co-trend hesitant fuzzy decision matrix to obtain the weighted co-trend hesitant fuzzy decision matrix:

$$\tilde{A}=\bar{A}•W=[\begin{array}{ccc} h\left(0.095,0.053,0.002\right) & h\left(0.050,0.051,0.117\right) & h\left(0.110,0.113,0.113\right)\\ h\left(0.099,0.053,0.053\right) & h\left(0.036,0.044,0.060\right) & h\left(0.028,0.077,0.088\right)\\ h\left(0.102,0.049,0.024\right) & h\left(0.042,0.099,0.144\right) & h\left(0.059,0.059,0.059\right)\\ h\left(0.074,0.074,0.074\right) & h\left(0.132,0.144,0.144\right) & h\left(0.030,0.090,0.095\right) \end{array}\begin{array}{cc} h\left(0.297,0.297,0.297\right) & h\left(0.032,0.070,0.151\right)\\ h\left(0.043,0.249,0.357\right) & h\left(0.041,0.159,0.159\right)\\ h\left(0.133,0.421,0.421\right) & h\left(0.125,0.141,0.151\right)\\ h\left(0.241,0.310,0.391\right) & h\left(0.047,0.075,0.156\right) \end{array}]$$

### 3.4. Determine the Relative Closeness between the Alternative to the Positive Ideal Solution

We determine the hesitation fuzzy positive ideal solution and the hesitation fuzzy negative ideal solution and use the hesitation fuzzy Euclidean distance to calculate the distance between each alternative and the hesitation fuzzy positive ideal solution and the hesitation fuzzy negative ideal solution. Then determine the relative closeness of each option to the positive ideal solution of the hesitation fuzzy.

First, we determine the hesitation fuzzy positive ideal solution $x^{+}$ and the hesitation fuzzy negative ideal solution $x^{-}$:

$$x^{+}=[\begin{array}{ccc} h\left(0.102,0.074,0.074\right) & h\left(0.132,0.144,0.144\right) & h\left(0.110,0.113,0.113\right) \end{array}\;\begin{array}{cc} h\left(0.297,0.421,0.421\right) & h\left(0.125,0.159,0.159\right) \end{array}]$$

(13) $$x^{-}=[\begin{array}{ccc} h\left(0.074,0.049,0.002\right) & h\left(0.036,0.044,0.060\right) & h\left(0.028,0.059,0.059\right) \end{array}\;\begin{array}{cc} h\left(0.043,0.249,0.297\right) & h\left(0.032,0.070,0.151\right) \end{array}]$$

Then, we calculate the Euclidean distance $D\left(x_{i},x^{+}\right)$ and $D\left(x_{i},x^{-}\right)$ between each option and the positive ideal solution and the negative ideal solution:

$$D\left(x_{1},x^{+}\right)=0.13212\;D\left(x_{2},x^{+}\right)=0.09697\;D\left(x_{3},x^{+}\right)=0.05714\;D\left(x_{4},x^{+}\right)=0.05003$$

(14) $$D\left(x_{1},x^{-}\right)=0.07453\;D\left(x_{2},x^{-}\right)=0.03273\;D\left(x_{3},x^{-}\right)=0.07262\;D\left(x_{4},x^{-}\right)=0.07589$$

Finally, we calculate the relative closeness of each option to the positive ideal solution of the hesitant fuzzy. Let $C_{i}$ denote the relative closeness between the option $x_{i}$ and the hesitation fuzzy positive ideal solution $x^{+}$, then:

$$C_{i}=\frac{D\left(x_{i},x^{-}\right)}{D\left(x_{i},x^{+}\right)+D\left(x_{i},x^{-}\right)}$$

The relative closeness of each option $x_{i}\left(i=1,2,3,4\right)$ to the positive ideal solution of the hesitant fuzzy is:

$$C_{1}=0.36066\;C_{2}=0.25235\;C_{3}=0.55965\;C_{4}=0.60268$$

According to the relative closeness between each option and the hesitant fuzzy positive ideal solution, all options are ranked:

$$x_{4}≻x_{3}≻x_{1}≻x_{2}$$

### 3.5. Uncertainty Analysis and Determination of Optimal Evaluation Results

Uncertainty analysis is the estimation and research on the changes and influences of various external factors that cannot be controlled beforehand in the production and operation process. We estimate the joint probability density function of the respond ability and environmental state of the enterprise based on the marketing data of the enterprise over the years and set the probability joint density function of response ability $Z_{x}$ and environmental state $H_{y}$ of each supplier:

$$f_{i}\left(D_{s},H_{y}\right),\left(i=1,2,3,4\right).\;f_{1}\left(Z_{x},H_{y}\right)=\{\begin{array}{c} 9e^{-\left(x+y\right)},x\geq 0,y\geq 0\\ 0,\mathrm{others} \end{array},\;f_{2}\left(Z_{x},H_{y}\right)=\{\begin{array}{c} e^{-\left(x+y\right)},x\geq 0,y\geq 0\\ 0,\mathrm{others} \end{array}\;f_{3}\left(Z_{x},H_{y}\right)=\{\begin{array}{c} 6e^{-\left(x+y\right)},x\geq 0,y\geq 0\\ 0,\mathrm{others} \end{array},\;f_{4}\left(Z_{x},H_{y}\right)=\{\begin{array}{c} 8e^{-\left(x+y\right)},x\geq 0,y\geq 0\\ 0,\mathrm{others} \end{array}$$

then:

$$f_{1}\left(Z_{x},H_{y}\right)=f\left(Z_{x}\right)f\left(H_{y}\right)=9e^{-x}•e^{-y},\;f_{2}\left(Z_{x},H_{y}\right)=f\left(Z_{x}\right)f\left(H_{y}\right)=e^{-x}•e^{-y}\;f_{3}\left(Z_{x},H_{y}\right)=f\left(Z_{x}\right)f\left(H_{y}\right)=6e^{-x}•e^{-y},\;f_{4}\left(Z_{x},H_{y}\right)=f\left(Z_{x}\right)f\left(H_{y}\right)=8e^{-x}•e^{-y}\;f_{i}\left(Z_{x}\right)=\int_{-\infty }^{+\infty }f_{i}\left(\frac{Z_{x}}{H_{y}}\right)f_{i}\left(H_{y}\right)dH$$

Then the probability of the enterprise affected by the environment can be approximately expressed as:

$$p_{i}=p_{i}\left(Z_{x}>Z_{a}\right)=\int_{Z_{a}}^{+\infty }\left[\int_{-\infty }^{+\infty }f_{i}\left(\frac{Z_{x}}{H_{y}}\right)f_{i}\left(H_{y}\right)dH_{y}\right]dZ_{y}\;=\int_{0}^{+\infty }\left[\int_{Z_{a}}^{+\infty }f_{i}\left(\frac{Z_{x}}{H_{y}}\right)dZ_{x}\right]f_{i}\left(H_{y}\right)dH_{y}$$

Supposing $Z_{a}=\frac{5}{2}$, $H^{*}=\frac{2}{3}$, $H^{**}=\frac{11}{2}$, we can get the following results:

$$p_{1}=0.37628,\;p_{2}=0.04181,\;p_{3}=0.25085,\;p_{4}=0.33447$$

We calculate the stability of the company in the environment according to $\left(1-p_{i}\right)$, and then reorder the solutions according to the relative size of $\left(1-p_{i}\right)•C_{i}$:

$$\left(1-p_{1}\right)•C_{1}=0.22495,\;\left(1-p_{2}\right)•C_{2}=0.24180,\;\left(1-p_{3}\right)•C_{3}=0.41926,\;\left(1-p_{4}\right)•C_{4}=0.40110\;x_{3}≻x_{4}≻x_{2}≻x_{1}$$

According to the size of $\left(1-p_{i}\right)•C_{i}$, the third supplier is the best supplier.

## 4. Discussion

In order to illustrate the effectiveness of this method, we compare it with other methods. First, we compare the weights determined by the “variance fluctuation” method in this paper with the weights determined by the method described in reference [44], as shown in Table 5. Then we compare the results of this paper with the results of the method in reference [44], as shown in Table 6.

Analyzing the attribute weights obtained by the above different methods, the attribute weights obtained in the reference [44] are more averaged and have little fluctuation; the weights determined in this paper based on the “variance fluctuation” method have relatively large fluctuations, the key points are more prominent and more effective, the importance of weight is reflected in the decision-making process. Therefore, the method in this paper is better than that in reference [44], The main reason is that reference [44] use the hesitation fuzzy distance measure to determine the weight of the attributes in the hesitation fuzzy environment. The element in the hesitation fuzzy number indicates that the decision maker is hesitant in several evaluation values when evaluating the degree $c_{j}\left(x_{i}\right)$ to which the option $x_{i}$ satisfies the attribute $c_{j}$. The greater the fluctuation of the evaluation value reflects the greater the psychological fluctuation of the decision maker, which reflects the importance of the attribute in some degree. Variance is an index used to measure the degree of volatility of a set of data, it can better reflect the psychological fluctuations of decision-makers in the decision-making process, which is in line with the fluctuations between elements in the hesitation fuzzy number. In this paper, in the method based on “variance fluctuation”, the particularity of hesitant fuzzy numbers is considered, so the result is more in line with the actual situation.

When the impact of environmental uncertainty is not considered, the method proposed in this paper is used to calculate the relative closeness of each option and the positive ideal solution. The best option obtained is the fourth supplier, which is the same as the results obtained by using the method described in reference [44], which proves the effectiveness of this method. When considering the impact of environmental uncertainty, the optimal option obtained by this method is the third supplier, which is different from the results obtained by the method in reference [44], which shows the necessity of considering the external environmental uncertainty. The main reason is the impact of attribute weight and environmental uncertainty, which also proves that this paper considers the uncertainty of external environment in supplier evaluation is more in line with the actual situation.

## 5. Conclusions

We propose a multi-attribute decision-making model in an uncertain environment, use hesitation fuzzy theory to solve the uncertainty of the environment, and according to the characteristics of the hesitation fuzzy number, a method suitable for the determination of the hesitation fuzzy number is proposed—the variance volatility determination method, and based on the theory of probability, an assessment method of environmental uncertainty is proposed, and finally we take supplier evaluation under an uncertainty environment as an example to illustrate the effectiveness of the model. The results show that: considering the uncertainty of the external environment, the original optimal supplier may not be the final optimal solution, especially under volatile economic and social conditions, and the impact of external environment uncertainty on enterprises is very important.

The research of this paper presented has a lot of practical and theoretical significance. In theory, the supply chain of an enterprise is a chain composed of many suppliers, and the quality of one supplier will affect the overall benefit of the whole supply chain. Therefore, a more effective supplier evaluation method is particularly important. A practical green supplier evaluation method can enrich the system of green supplier evaluation methods, promote the progress of green supplier evaluation methods and bring more alternative methods for green supplier evaluation. In practice, with the enhancement of global environmental awareness, companies are facing more stringent environmental requirements. Considering environmental factors in the process of supply chain evaluation can have a more positive impact on the whole supply chain of enterprises, bring good economic and environmental benefits to enterprises, enhance their competitive advantages and determine their competitiveness in the future.

The difficulty of uncertainty evaluation is finding the appropriate evaluation factors, collecting data based on the evaluation factors and obtaining the joint probability density function, besides, the assessment of corporate respond ability and load ability is also an important task. In this process, the determination of the probability density function is a very critical step. Determining the evaluation indicators of respond ability and load ability of an enterprise in different environments is the focus and difficulty of future research. Besides, the determination of the joint probability density function of environmental state and respond ability is also an important task. In the future, hesitant fuzzy method can be extended to more application fields, such as safety risk assessment, quality evaluation of brain hemorrhage treatment, etc. [57].

## Figures and Tables

**Table 1:** Supplier Evaluation factor system.

Symbol	Factor	Detailed Description
$C_{1}$	Cost	Product price, product cost, transportation cost, product price stability, etc.
$C_{2}$	Quality	Quality certification, product qualification rate, quality management system, etc.
$C_{3}$	Innovation	New product R & D capability, R & D funding level, etc.
$C_{4}$	Environment	Pollution control, resource recycling rate, environmental protection level of products in life cycle, etc.
$C_{5}$	Compatibility	Compatibility of management system, compatibility of cultural level, compatibility of business philosophy, etc.

**Table 2:** Hesitation Fuzzy Decision Matrix of Suppliers.

	$C_{1}$	$C_{2}$	$C_{3}$	$C_{4}$	$C_{5}$
$X_{1}$	(0.28, 0.60, 0.98)	(0.78, 0.33, 0.34)	(0.90, 0.87)	(0.69)	(0.43, 0.20, 0.93)
$X_{2}$	(0.60, 0.25)	(0.24, 0.29, 0.40)	(0.70, 0.22, 0.61)	(0.10, 0.58, 0.83)	(0.25, 0.98)
$X_{3}$	(0.63, 0.23, 0.82)	(0.66, 0.96, 0.28)	(0.47)	(0.98, 0.31)	(0.77, 0.93, 0.87)
$X_{4}$	(0.44)	(0.96, 0.88)	(0.24, 0.75, 0.72)	(0.91, 0.56, 0.72)	(0.29, 0.46, 0.96)

**Table 3:** Standardized Hesitant Fuzzy Decision Matrix.

	$C_{1}$	$C_{2}$	$C_{3}$	$C_{4}$	$C_{5}$
$X_{1}$	(0.28, 0.60, 0.98)	(0.33, 0.34, 0.78)	(0.87, 0.90, 0.90)	(0.69, 0.69, 0.69)	(0.20, 0.43, 0.93)
$X_{2}$	(0.25, 0.60, 0.60)	(0.24, 0.29, 0.40)	(0.22, 0.61, 0.70)	(0.10, 0.58, 0.83)	(0.25, 0.98, 0.98)
$X_{3}$	(0.23, 0.63, 0.82)	(0.28, 0.66, 0.96)	(0.47, 0.47, 0.47)	(0.31, 0.98, 0.98)	(0.77, 0.87, 0.93)
$X_{4}$	(0.44, 0.44, 0.44)	(0.88, 0.96, 0.96)	(0.24, 0.72, 0.75)	(0.56, 0.72, 0.91)	(0.29, 0.46, 0.96)

**Table 4:** Co-trend hesitant fuzzy decision matrix.

	$C_{1}$	$C_{2}$	$C_{3}$	$C_{4}$	$C_{5}$
$X_{1}$	(0.72, 0.40, 0.02)	(0.33, 0.34, 0.78)	(0.87, 0.90, 0.90)	(0.69, 0.69, 0.69)	(0.20, 0.43, 0.93)
$X_{2}$	(0.75, 0.40, 0.40)	(0.24, 0.29, 0.40)	(0.22, 0.61, 0.70)	(0.10, 0.58, 0.83)	(0.25, 0.98, 0.98)
$X_{3}$	(0.77, 0.37, 0.18)	(0.28, 0.66, 0.96)	(0.47, 0.47, 0.47)	(0.31, 0.98, 0.98)	(0.77, 0.87, 0.93)
$X_{4}$	(0.56, 0.56, 0.56)	(0.88, 0.96, 0.96)	(0.24, 0.72, 0.75)	(0.56, 0.72, 0.91)	(0.29, 0.46, 0.96)

**Table 5:** Weight comparison of different decision-making methods.

Weights Comparison		
	Article	Reference [44]
$C_{1}$	0.132	0.137
$C_{2}$	0.150	0.271
$C_{3}$	0.126	0.203
$C_{4}$	0.430	0.186
$C_{5}$	0.162	0.203

**Table 6:** Result comparison of different decision-making methods.

Result Comparison		
	Article	Reference [44]
$X_{1}$	0.22495	−0.38433
$X_{2}$	0.24180	−1.03775
$X_{3}$	0.41926	−0.09091
$X_{4}$	0.40110	0.24519

## Data Availability

Not applicable.
